# Comparative study of substrate free and amniotic membrane scaffolds for cultivation of limbal epithelial sheet

**DOI:** 10.1038/s41598-018-32914-0

**Published:** 2018-10-02

**Authors:** Hyun Jung Lee, Sang Min Nam, Sae Kyung Choi, Kyoung Yul Seo, Hyun Ok Kim, So-Hyang Chung

**Affiliations:** 1 Department of Biochemical Engineering, Seoil University, Seoul, Korea; 2 Department of Ophthalmology, CHA Bundang Medical Center, CHA University, Seongnam, Korea; 3 Department of Obstetrics and Gynecology, Incheon St. Mary’s Hospital, Incheon, Korea; 4 Department of Ophthalmology, Institute of Vision Research, Yonsei University College of Medicine, Seoul, Korea; 5 Department of Laboratory Medicine, Yonsei University College of Medicine, Seoul, Korea; 6 Department of Ophthalmology and Visual Science, Catholic Institute of Visual Science, College of Medicine, The Catholic University of Korea, Seoul, Korea

## Abstract

Transplantation of cultivated limbal epithelial transplantation has been proven to restore the corneal surface in limbal stem cell deficiency (LSCD). Here we comparatively investigated the optimized conditions and the efficiency of limbal epithelial sheet growth in three media conditions as well as with substrate free (transwell), human amniotic membrane (HAM) sutured onto transwell inserts (HAMTW), and HAM slide scaffold (HAMS). Outcomes evaluated were outgrowth sheet size from limbal explants, expression of stem/progenitor cell markers p63α, ABCG2 and CK15, and colony formation efficiency (CFE). Additionally, limbal epithelial sheets on HAMS were transplanted into corneas of LSCD rabbit models. Limbal epithelial sheets with 5% human AB serum showed the greatest increase in ABCG2 efflux activity (JC1^low^), p63α expression, and CFE compared in both conditions without HAM and with HAM, respectively. The outgrowth sheet size, cell yield, and Ki67 expression were increased in limbal epithelial sheets on HAMS compared to transwell and HAMTW. ABCG2 efflux activity, p63α and CK15 expressions, and CFE were also increased in limbal epithelial sheets on HAMS as well. In corneas of transplanted rabbit LSCD models, p63α expressions were noted in the basal layers and CK12 expressions were observed in superficial layers. Cultivation of limbal epithelial sheet on HAMS with xeno-free medium enhances the growth and stemness of limbal epithelial sheets.

## Introduction

Limbal stem cells (LSCs) are essential to maintain cornea epithelium and endowed with a capacity for self-renewal and extended proliferative potential^1,2^. Dysfunction or loss of these cells by chemical or thermal injury, radiation, repeated surgical interventions, and immunological disorders, results in limbal stem cell deficiency (LSCD). This is characterized by loss of corneal clarity, conjunctivalization, and visual loss^3^.

Transplantation of cultivated limbal epithelial sheets has proven to be effective to restore healthy corneal epithelium and vision^4–9^. Several substrates have been utilized to culture limbal epithelial cells such as human amniotic membranes (HAM)^4^, fibrin gels^10^, plasma polymer-coated surfaces^11^, biodegradable matrices^12^, temperature-responsive culture dishes^13^, or feeder layers of mouse NIH 3T3 cells^7^.

Even though native and the synthetic materials would completely avoid donor tissue-derived infection, HAM expresses several factors that promote epithelial growth and suppress surgery-induced inflammation and corneal stroma scar^14,15^; also the hypoimmunogenic property of HAM helps reduce immunologic problems during transplantation^16^.

Failure of limbal epithelial sheet transplantation may relate to the depletion of LSCs in culture^17^. Past studies demonstrated that the favorable outcome of early limbal epithelial sheet transplantation had a high correlation with expression of p63, a protein essential to maintaining the proliferative potential of epithelial cells of ectodermal origin and associated with the level of stemness in the limbal-corneal epithelial lineage grafted population^8^. Thus, investigation of optimal culture conditions for increased percentile of stem/progenitor cells with regenerative capacity within limbal epithelial sheets is critically important to treat LSCD. Toward this goal, we comparatively investigated the proliferation potential and stemness of limbal epithelial sheets on substrate free and amniotic membrane scaffolds with xenobiotic free medium and demonstrated limbal epithelial sheet on HAM slide scaffold (HAMS) enhances the growth and stemness of limbal epithelial sheets.

## Results

### Effects of culture media on the growth and the activation of limbal stem/progenitor cells in limbal epithelial outgrowth sheets

**Figure 1:**
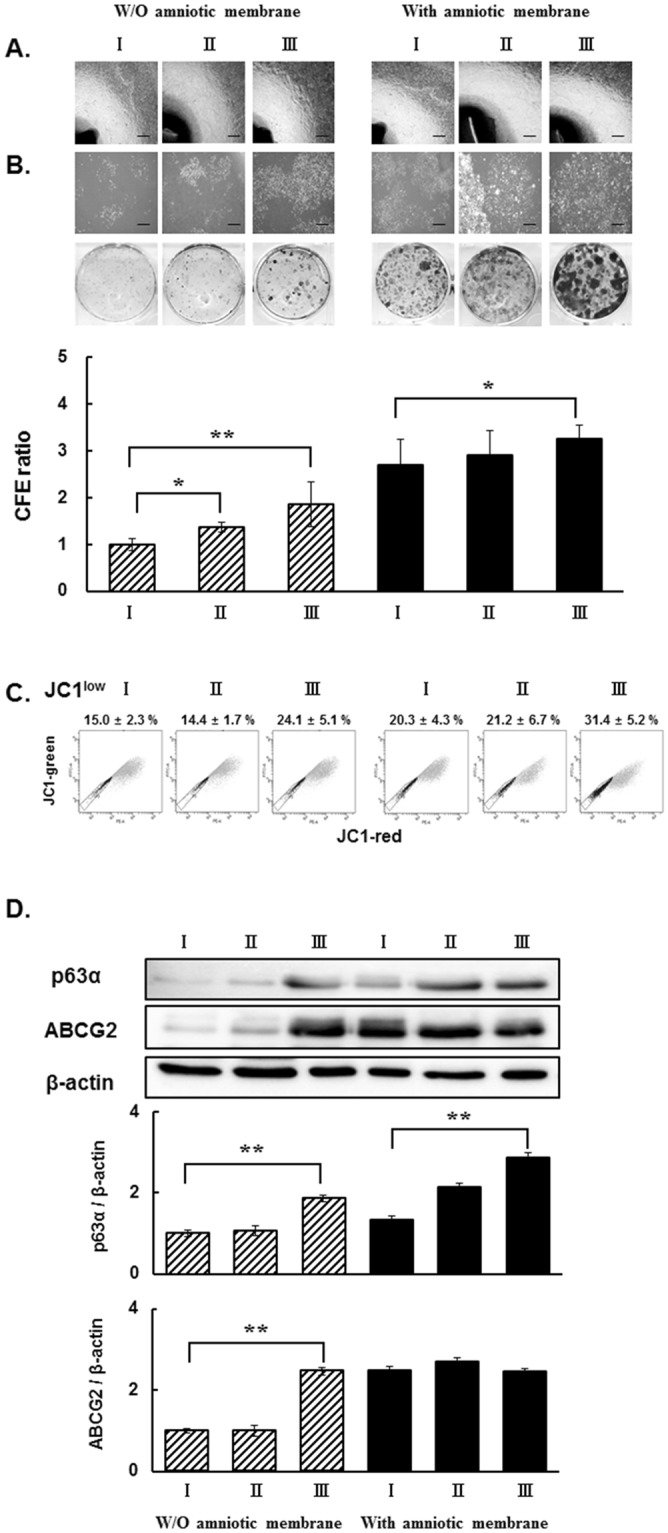
Effects of culture media on activation of limbal stem/progenitor cells in limbal explant outgrowth sheets. (**A**) Representative phase contrast micrographs of limbal explant and outgrowths in culture medium complemented with (I) 5% FBS with CT, (II) 5% FBS without CT, and (III) 5% human serum without CT using transwell (without; W/O amniotic membrane) and amniotic membrane. Bar = 50 µm. (**B**) Representative phase contrast micrographs of holoclone colonies from outgrown cells from limbal explant (upper). Photographs of colonies stained with Coomassie blue R solution (lower) and CFE ratio. (**C**) Representative JC1 staining bivariate plots of limbal explant outgrowth cultures with JC1 exclusion cell cohort (JC1^low^) indicated. Mean ± SD for three independent experiments are shown within the plots. **(D**) Representative western blot image on p63α and ABCG2 protein expression. All signal intensities were normalized to the signal generated in the same sample by β-actin. *p < 0.05, **p < 0.01 (n = 6 from 3 donors).

To select the best culture media, we compared the following three conditions, (I) supplemented hormonal epithelial medium (SHEM) medium including 5% fetal bovine serum (FBS) with cholera toxin (CT), (II) 5% FBS without CT, and (III) 5% human serum without CT on transwell (TW) and HAMS. After 4 days, the cultures displayed visible cell outgrowths surrounding explant in all six conditions (Fig. 1A). After 12 days, isolated cells were seeded at clonogenic density in CNTP medium. All colonies were holoclones^18^. As shown in Fig. 1B, condition III on TW had a significant increase in colony formation efficiency (CFE) (III/I CFE ratio = 1.8 ± 0.47, p < 0.01; II/I CFE ratio = 1.4 ± 0.10, p < 0.05). CFE ratio in condition III was also the highest (III/I CFE ratio = 1.2 ± 0.29, p < 0.05) in limbal outgrowth cells on HAMS. As shown in Fig. 1C, respective to (I), outgrowth sheets in 5% human serum without CT (III) induced an 9.08% increase in JC1^low^ (p < 0.01), while 5% FBS without CT (II) elicited no difference in percentage of cells excluding JC1 which indicated ABCG2 efflux activity of limbal stem/progenitor cells on TW. JC1^low^ exclusion percentages of all three conditions on HAM were maintained above 20.3% and those in condition III induced a more than 8% increase in JC1^low^ compared to two other conditions. Western blot analysis revealed statistically significant increases in two limbal stem/progenitor cell markers, unique C-termini α isoform of tumor protein p63 (p63α) and ATP-binding cassette sub-family G member 2 (ABCG2) of limbal explant outgrowth sheets carried out in condition III on TW (Fig. 1D, III/I in p63α expression = 1.8 ± 0.07, p < 0.01; III/I in ABCG2 expression = 2.4 ± 0.10, p < 0.01), and increase in p63α expression on HAMS (III/I = 2.1 ± 0.09, p < 0.01) (Fig. 1D). Therefore, 5% human serum without CT (III) condition on both TW and HAMS, which is xenofree and the best condition for the activation of stem/precursor properties of limbal explant outgrowth populations, was adopted as the media for further experiments.

### Effects of HAMS on the growth and proliferation of limbal epithelial outgrowth cells from explants

**Figure 2:**
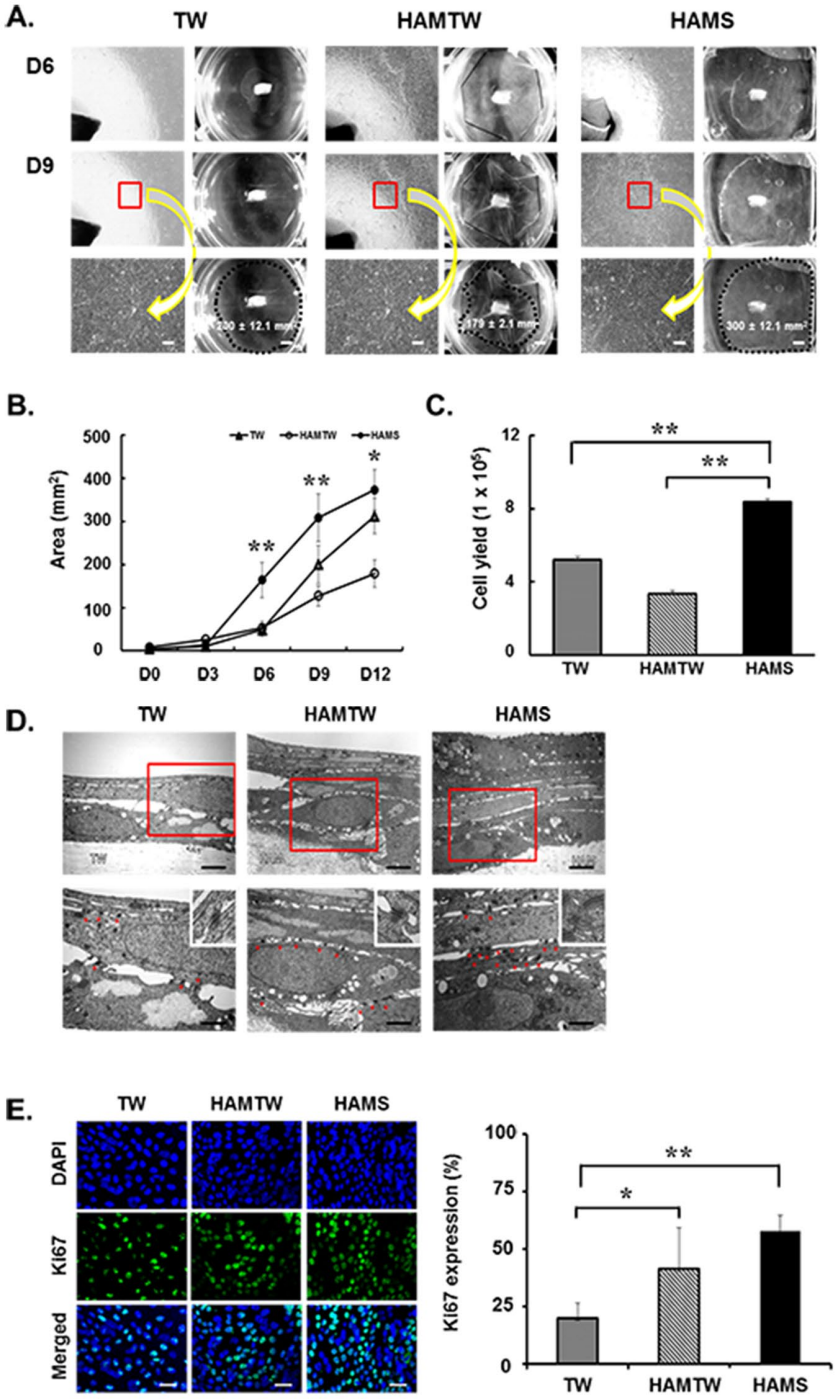
Limbal explant outgrowth sheets on substrate free, HAMTW, and HAMS. (**A**) Representative phase contrast micrographs of limbal explant outgrowth sheets on days 6 and 9 in TW, HAMTW, and HAMS. Bar = 50 µm. Mean ± SD for six independent experiments from three donors are shown. (**B**) Area of limbal explant outgrowth sheets by day 12. (**C**) Cell yields after culture for 12 days. (**D**) Ultrastructure of limbal explant outgrowth sheets using transmission electron microscopy. The multi-layers (red insert) shown on top were observed under different magnifications on the bottom, respectively. The red stars demonstrated cell-cell junction spaces which were magnified on the top right of the pictures (bottom). Bar = 2 µm (upper) and 0.2 µm (lower). (**E**) Representative images of explant outgrowths stained for Ki67 and graph for Ki67 expressions. Bar = 20 µm. *p < 0.05, **p < 0.01 (n = 6 from 3 donors).

Limbal epithelial outgrowth size from explant on the HAMS was definitely larger by day 6 compared to those under the other two conditions. On day 9, limbal explant outgrowth size on HAMS was 300 ± 12.1 mm^2^, which was significantly larger than those on TW (200 ± 12.1 mm^2^, p < 0.05) or human amniotic membrane sutured onto Transwell inserts (HAMTW, 179 ± 2.1 mm^2^, p < 0.01) (Fig. 2A). Limbal epithelial outgrowth sheet on HAMTW showed irregular shapes on wrinkled HAM because of poor mechanical support from sutured HAM onto TW. From days 6 to 12, the outgrowth size was significantly larger on the HAMS compared to other two conditions (Fig. 2B, p < 0.01). Interestingly, limbal outgrowth size on HAMTW was significantly smaller compared to TW. When cells were harvested for downstream processing and counted, the yields for HAMS were significantly higher than those from the other two conditions (Fig. 2C, p < 0.01).

On transmission electron microscopy (TEM) analysis, limbal epithelial outgrowth sheets on HAMTW and HAMS consisted of 3–6 cell layers with attached to the HAM, which showed flat superficial cells and columnar/cuboidal shaped basal cells. But only one to two layers was observed in sheets on TW (Fig. 2D). The limbal epithelial outgrowth sheets on HAMTW and HAMS had more closed cell-cell junction space than on TW (Fig. 2D, upper). Although cells in all three conditions had numerous desmosomes, those on HAMS had more desmosomes than in the other two conditions (Fig. 2D, lower).

To confirm the increased proliferation potential of limbal epithelial outgrowth cells on HAMS, Ki67, a proliferation marker, was evaluated. Immunostaining demonstrated more Ki67-positive cells in HAMS than the other two conditions. The quantification of immunostaining for Ki67 revealed that Ki67-positive cells was significantly increased in both HAMTW and HAMS by 2–3 folds relative to the control (Fig. 2E, HAMTW/TW Ki67 expression = 2.0 ± 0.89, p < 0.05; HAMS/TW Ki67 expression = 2.9 ± 0.33, p < 0.01). These results indicate that limbal explant outgrowth cells on HAMS showed fast growth and had extended proliferative potential.

### Effects of HAMS on the activation of limbal stem/progenitor cells in limbal epithelial outgrowth s**h**eets

**Figure 3:**
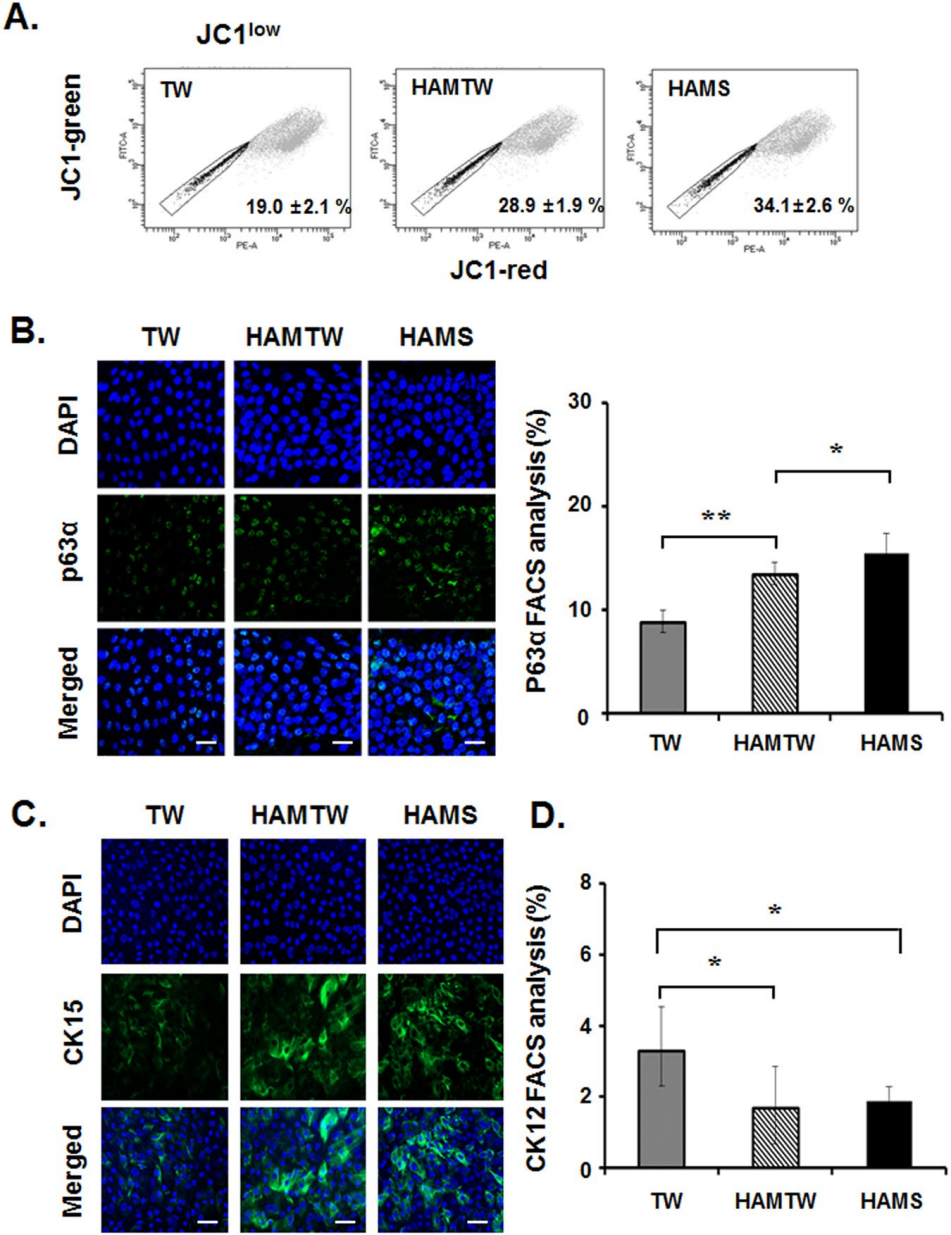
Preservation effects of stem/precursor properties in limbal epithelial outgrowth sheets on substrate free, HAMTW, and HAMS. (**A**) JC1_low_ is indicated. Mean ± SD for 6 independent experiments are shown within the plots. (**B**) Representative images of outgrowth sheets stained for p63α and p63α^+^ cells from FACS analysis were shown. (**C**) Representative images of outgrowth sheets stained for CK15. Bar = 20 µm. (**D**) FACS analysis of CK12^+^ cells expression were shown. *p < 0.05, **p < 0.01 (n = 6 from 3 donors).

To expand the phenotypic characterization of the effect of HAMS, we investigated the expression of limbal stem/progenitor cell markers, ABCG2, p63α, cytokeratin 15 (CK15) and the corneal epithelial differentiation marker cytokeratin 12 (CK12). Consistent with the concept that JC1^low^ is an ABCG2 substratum, limbal epithelial sheet on HAMS had greater percentages of cells resisting staining by JC1 compared with sheets grown under the other two conditions, respectively (p < 0.01) (JC1^low^, Fig. 3A).

Transcription factor p63 and in particular the isoform ΔNp63α has been linked to stemness and success of early limbal epithelial sheet transplantation. Immunostaining showed that the target epitope was localized exclusively to the nucleus (Fig. 3C) as expected for the truncated form of p63 (ΔNp63) present in epithelia and a substantial increase in p63α stained cells in outgrowth cells on HAMS compared to TW. Also, FACS analysis showed significantly more p63α^+^ cells in limbal outgrowth cells on HAMS (15.5 ± 1.9%) compared to those on TW (8.8 ± 1.2%; p < 0.01) and HAMTW (13.5 ± 1.9%; p < 0.01) (Fig. 3B). As shown in Fig. 3C, immunostaining demonstrated more CK15^+^ cells in outgrowth cells on HAMS than other two conditions. For the corneal epithelial differentiation marker CK12, immunostaining did not demonstrate any CK12^+^ cell in all three conditions (data not shown). FACS analysis showed significantly lower CK12^+^ cells in limbal outgrowth cells on HAMTW (1.7 ± 1.17%) and HAMS (1.9 ± 0.42%) than on TW (3.3 ± 1.23%) (p < 0.05) (Fig. 3D).

**Figure 4:**
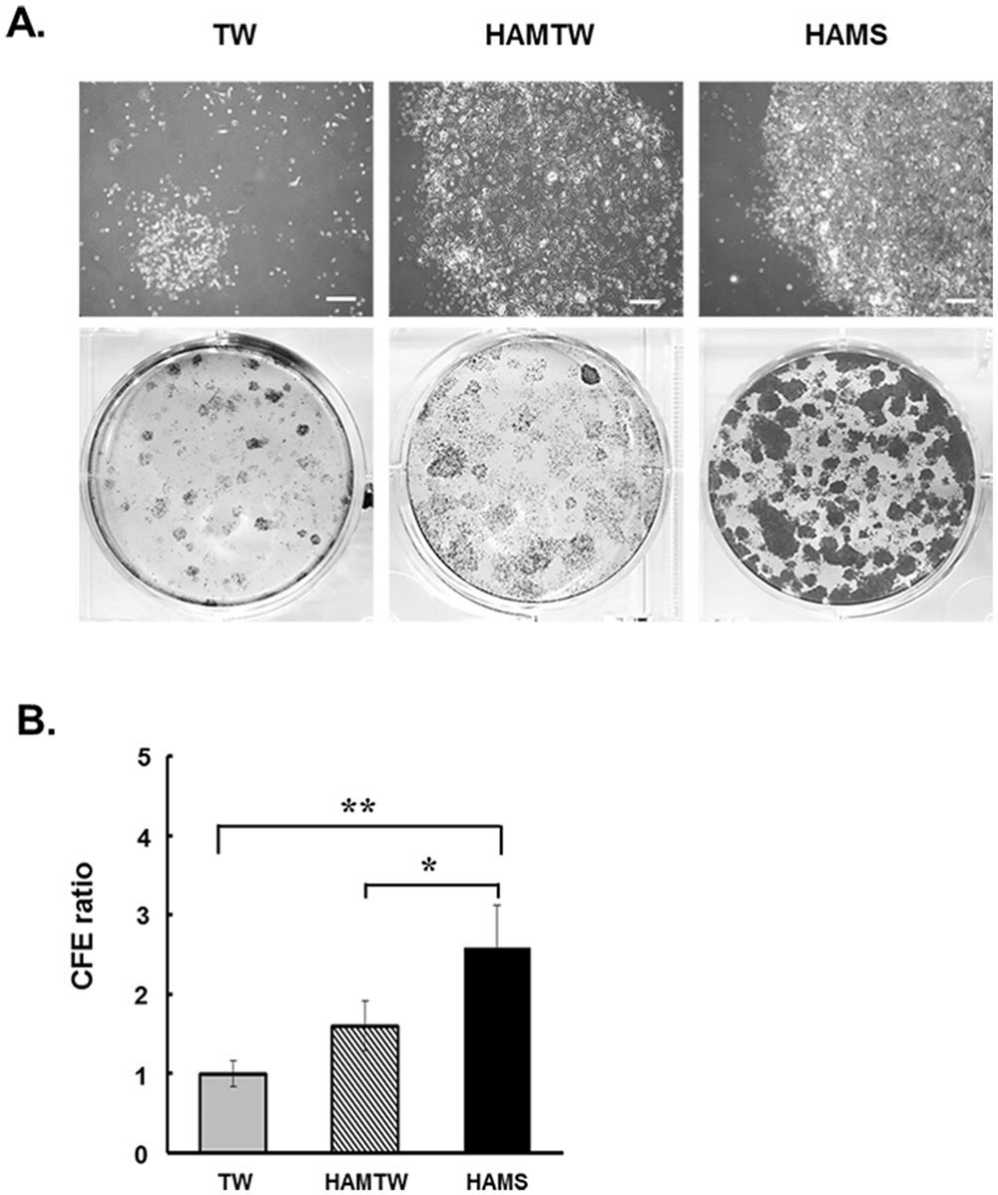
CFE from limbal explant outgrowth sheets cells on substrate free, HAMTW, and HAMS. (**A**) Phase contrast micrographs and Coomassie blue stained holoclone colonies from limbal explant outgrowth sheets in CNTP media. (**B**) CFE ratio normalized to TW. Data are from six independent experiments. *p < 0.05, **p < 0.01 (n = 6 from 3 donors).

Finally, we investigated the effect of the HAMS on the preservation of clonogenic capacity in the limbal outgrowth populations. Cells from HAMS showed a higher holoclone clonogenic capacity than those from other conditions (HAMS/TW CFE ratio = 2.4 ± 0.06, p < 0.01; HAMS/HAMTW CFE ratio = 1.5 ± 0.06, p < 0.05) (Fig. 4). These results collectively suggest that the limbal epithelial sheet on HAMS preserve a significant number of effective limbal stem/progenitor cells than those on TW or HAMTW.

### Outcomes of transplantation of limbal epithelial sheets on HAMS into rabbit LSCD models

**Figure 5:**
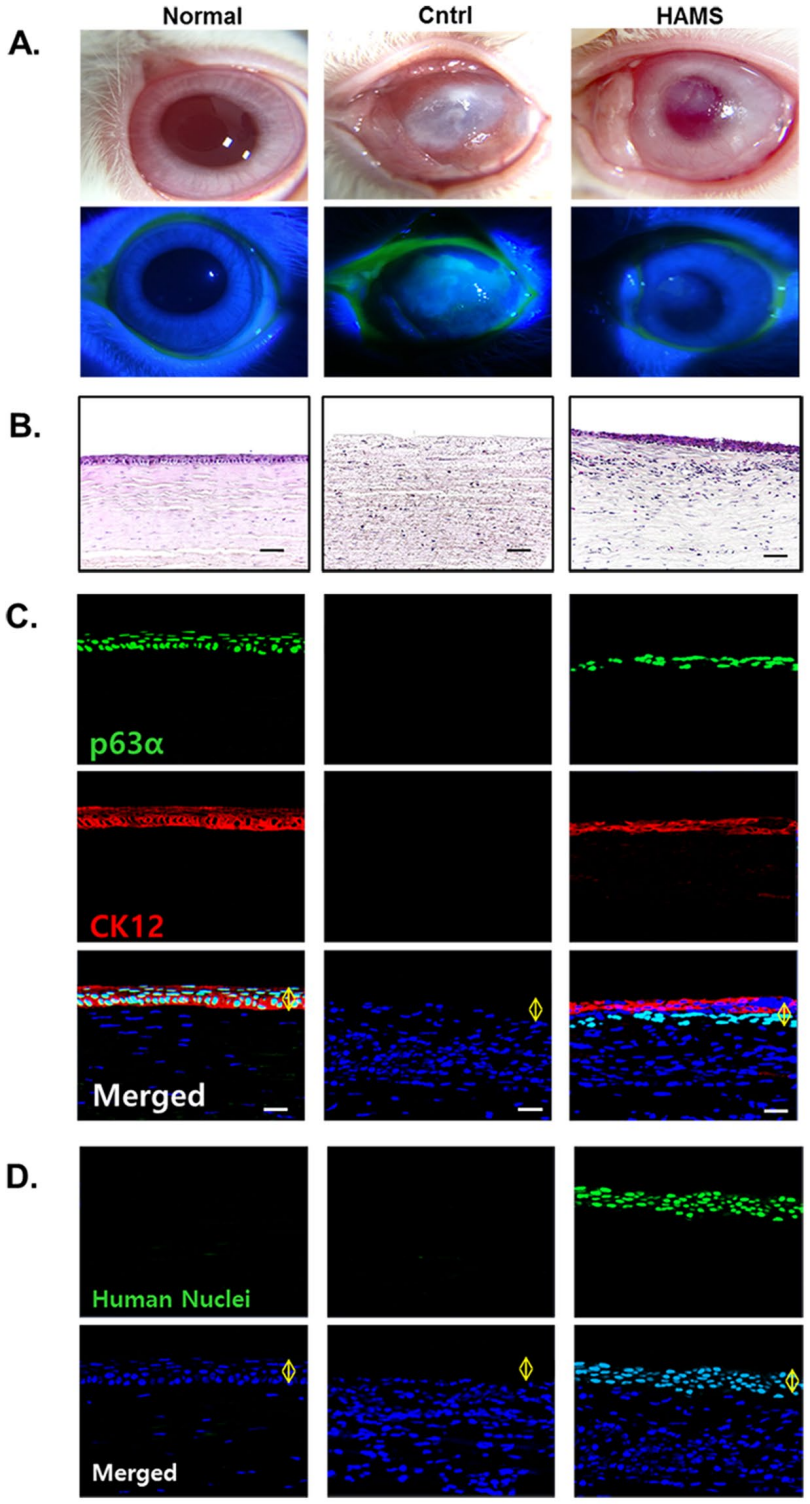
Results of transplantation of human limbal explant outgrowth sheets on HAMS into rabbit LSCD models. (**A**) Microscopic photos of rabbit ocular surface at 4 weeks after transplantation of limbal explant outgrowth sheets onto HAMS. (**B**) Hematoxylin-eosin stained photos of rabbit corneas at 4 weeks after transplantation. Bar = 50 µm. (**C**) Immunohistochemical examination of rabbit corneas for p63α and CK12 at 4 weeks after transplantation. Bar = 20 µm. (**D**) Immunohistochemical examination for human nuclei. Bar = 20 µm. Normal; healthy rabbit cornea, Cntrl: corneas of LSCD models without transplantation, HAMS: corneas of LSCD models with transplantation of humal limbal outgrowth sheets on HAMS.

As we found limbal outgrowth sheets on HAMS preserved better stemness on those on TW or HAMTW, we transplanted limbal epithelial sheets on HAMS onto corneas of rabbits LSCD models. During the follow-up, the sheets on the corneal surface were intact without any dislodgement. The transplanted corneal surfaces did not show epithelial defects for 4 weeks. In comparison, eyes treated identically but without transplantation (control) showed cornea opacity and non-healed corneal epithelial defect (Fig. 5A).

At postoperative 4 weeks, histology of rabbit cornea demonstrated typical appearance of corneal epithelium with flat superficial cells and cuboidal basal cells similar as normal rabbit cornea (Fig. 5B). However, in the control group, corneal epithelial layers could not be observed. Immunohistochemistry of rabbit cornea demonstrated that the basal cell layer expressed p63α intensively and superficial layers expressed CK12 (Fig. 5C). Rabbit central cornea transplanted with human limbal epithelial sheet on HAMS showed positive stains for the anti-human nuclei antibody throughout all layers which indicate epithelium in the rabbit corneas were came from the transplanted human limbal epithelial sheets not rather than zonal repopulations of remained rabbit limbal stem cells (Fig. 5D).

## Discussion

Transplantation cultivated limbal epithelial sheet has been considered as one of the standard treatments in LSCD^6,7,19–22^. However, the protocol for cultivation of limbal epithelial cells differs significantly. Protocols include different culture technique such as isolated epithelial cell cultures or limbal epithelial sheet generation from limbal explants, the application of the mouse 3T3 feeder cell layer, and different substrates^6,7,20,21,23–25^.

The slow cycling status of LSCs is abrogated during wound healing to provide the extra transient amplifying cells needed to rapidly close a wound. There is sound evidence that during such temporary events, whether *in vivo*^26^ or *ex vivo*^27^, LSCs multiply in such a manner that cells displaying stem/precursor cell features undergo a large expansion within the limbal niche and, subsequently, in outgrowth cells from limbal explants^27^. This property underpins the ability of limbal explant outgrowth sheets, the most common transplant approach for LSCD, to restore the health of the eye afflicted by this deficiency^8,20,21,23^.

With regard to culture medium, SHEM including FBS and CT has been published as a standard medium for limbal outgrowth culture from explant^28,29^. FBS has been considered a key component to achieve successful cell cultures^25^ and CT has been reported to stimulate colony growth from cultured human epidermal keratinocytes^30^ and limbal epithelail cells^31^. For clinical application, we compared SHEM in FBS with CT or without CT because CT is a protein complex secreted by the bacterium *Vibrio cholerae*, and has been reported to have side effects in humans^32^. In addition, we replaced 5% FBS with 5% clinical grade human serum because these animal products might possess a risk of spreading transmission of animal pathogens that may provoke xenogeneic reactions. Accordingly, a xenofree culture condition is essential for clinical application of human limbal outgrowth sheets and 1–10% human serum is reported to be appropriate for cultivation of human limbal epithelial cells^21,23,24,33^. Our results revealed SHEM in 5% human serum without CT had higher CFE, ABCG2 efflux activity (JC1^low^), and p63α expression in limbal outgrowth cells on TW and HAMS compared to SHEM in 5% FBS with or without CT. Therefore, we selected SHEM in 5% human serum without CT for further experiments with HAMS.

The key finding of this study is to use HAMS for limbal outgrowth culture from explant. HAM as a substrate for limbal epithelial culture has been proposed to be useful because it is a strongly biodegradable, hypoimmunogenic, and easily manipulated carrier. Until now the application of HAM as a substrate in clinical settings included HAM on the transwell insert^20,22,33,34^ or interlockable plastic amnion rings^21,35^. In this study, we designed HAMS in which HAM was spread and tucked around a glass slide tightly fit in a 35-mm^2^ dish. This HAM slide scaffold provided a smooth surface and mechanical tension for HAM, resulting in fast growth of limbal explant outgrowth sheets and higher cell yield compared to HAMTW. Limbal outgrowth size and cell yield on HAMTW was significantly smaller compared to those on TW without HAM because of poor mechanical support from sutured HAM onto TW. In addition, limbal epithelial sheets on HAMS contained more Ki67-positive cells, indicating higher proliferative potential than those on HAMTW. The key element of the success of transplantation is the regeneration capacity of limbal epithelial sheets. Rama *et al*. showed that the percentage of p63 bright cells is related to the success after transplantation, indicating that p63 expression levels correlate with the level of stemness in the limbal-corneal epithelial lineage grafted population^8^. They reported that cultures in which p63-bright cells constituted more than 3% of the total number of clonogenic cells were associated with successful transplantation in 78% of patients^8^. Interestingly, our limbal outgrowth cells on HAMS constituted more than 15% p63α^+^ cells of the total cells by FACS analysis, which was significantly higher compared to cells on TW and previous study by Rama *et al.*^8^. When we examined other limbal stem/progenitor cell markers, we found increased ABCG2-dependent dye exclusion activity (JC1^low^ % cells) and CK15 expressions in outgrowth cells on HAMS compared to those on HAMTW or TW. Lastly, colonogenic capacity was also significantly enhanced in limbal outgrowth cells on HAMS compared to those on HAMTW or TW. These results indicate that limbal epithelial sheet from explant on HAMS preserve higher stemness and clonogenic capacity than those on HAMTW or TW.

For clinical application of limbal epithelial cell cultures to LSCD patients, few previous studies demonstrated results of limbal explant culture on HAM in xenofree medium condition. Gonzalez *et al*. reported that for human limbal epithelial cultivation explant on HAM SHEM without CT and with 5% autologous serum was the most efficient and consistent in supporting the LSC phenotype and growth, which was similar as our medium condition^36^. On the contrary to this Luznik *et al*. demonstrated that limbal explant cultures on HAM sutured on TW and TW, using culture media with standard SHEM or SHEM with human serum, yield tissues with similar morphology, gene expression and keratin staining^37^. Our study revealed that limbal epithelial sheets from explant on HAMS in SHEM with human serum showed faster growth, higher CFE, ABCG2 efflux activity, and p63α expression compared to HAM sutured on TW or TW. Moreover, limbal epithelial sheet on HAMTW showed irregular shapes on wrinkled HAM because of poor mechanical support from sutured HAM onto TW. Therefore, we believe limbal epithelial sheet from explant on our new HAMS with higher regenerative capacity might be better option for clinical application.

To determine regeneration capacity of limbal epithelial sheets on HAMS, we transplanted sheets to the cornea of rabbit LSCD models. At 4 weeks after transplantation, the transplanted corneas had a typical corneal appearance with intensive p63α expression in the basal layer and CK12 expressions in superficial layers. These results suggest that limbal epithelial sheets on HAMS maintain corneal epithelial regenerative capacity after transplantation.

In conclusion, limbal epithelial sheet generation on HAM slide scaffold with xenofree medium enhances the growth and stemness of limbal epithelial sheets from explant culture. From a practical perspective, our results indicate that this culture protocol on HAMS could improve survival of limbal stem/progenitor cells and maintain corneal epithelial regeneration capacity after transplantation.

## Materials and Methods

### Tissue procurement

Post- keratoplasty discards of human corneal-limbal tissues from unidentifiable cadavers were obtained from Seoul St. Mary’s Hospital Eye Bank (Seoul, Korea). Use of these tissues was not considered as research on human by The Institutional Review Board. Tissue acceptance criteria were as follows (1) donor ages of 30 to 65 years at death; (2) tissue harvest occurring within 12 h of death; (3) tissue storage in Optisol (Bausch & Lomb, Rochester, NY) for less than 72 h after harvest, and (4) donor had testing negative for Epstein-Barr virus, human immunodeficiency virus, hepatitis B or C, and syphilis. The corneas obtained were split into 12 equal parts. After scleral and conjunctival tissues were trimmed, each corneal part was cut in limbal strips 0.5-mm-wide. Informed consent was procured from women before cesarean-section delivery when HAM was obtained and its use was approved by the Institutional Review Board of the College of Medicine, The Catholic University of Korea. Under sterile conditions, HAM was washed, placed over a nitrocellulose membrane, and preserved in tissue culture medium and glycerol (1:1 mix) and stored at −80 °C until further use. After thawing, HAM was treated with cold 5 M urea (Sigma) for 5 min and scraped delicately with a #15 blade to remove remaining epithelial cells. Completely de-epithelialized HAM was transferred onto Transwell or slide scaffold.

### Culture medium

The composition of SHEM is composed of 950 ml of a 1:1 mix of Dulbecco’s modified minimal essential medium and HAM F12 (DMEM/F-12, Gibco, Grand Island, NY), 50 ml of FBS, 0.5% dimethyl sulfoxide (Sigma), 5 µg of human recombinant epidermal growth factor (Sigma), 14 mg of O-phosphoethanolamine (TCI, Tokyo, Japan), 5 mg of ethanolamine (Sigma), 10 µg of CT (Sigma), 1 × insulin-transferrin-selenium (Gibco), and 1 × penicillin–streptomycin (Gibco) mixes (medium I; 5% FBS with CT)^28,29^. A single batch of FBS was used in all experiments. CT was excluded from medium II (5% FBS without CT). In medium III, medium I was altered by replacing FBS with 50 ml of human AB serum (Sigma) (5% human serum without CT).

### Cultivation of human limbal outgrowth sheets from explant on three conditions

**Figure 6:**
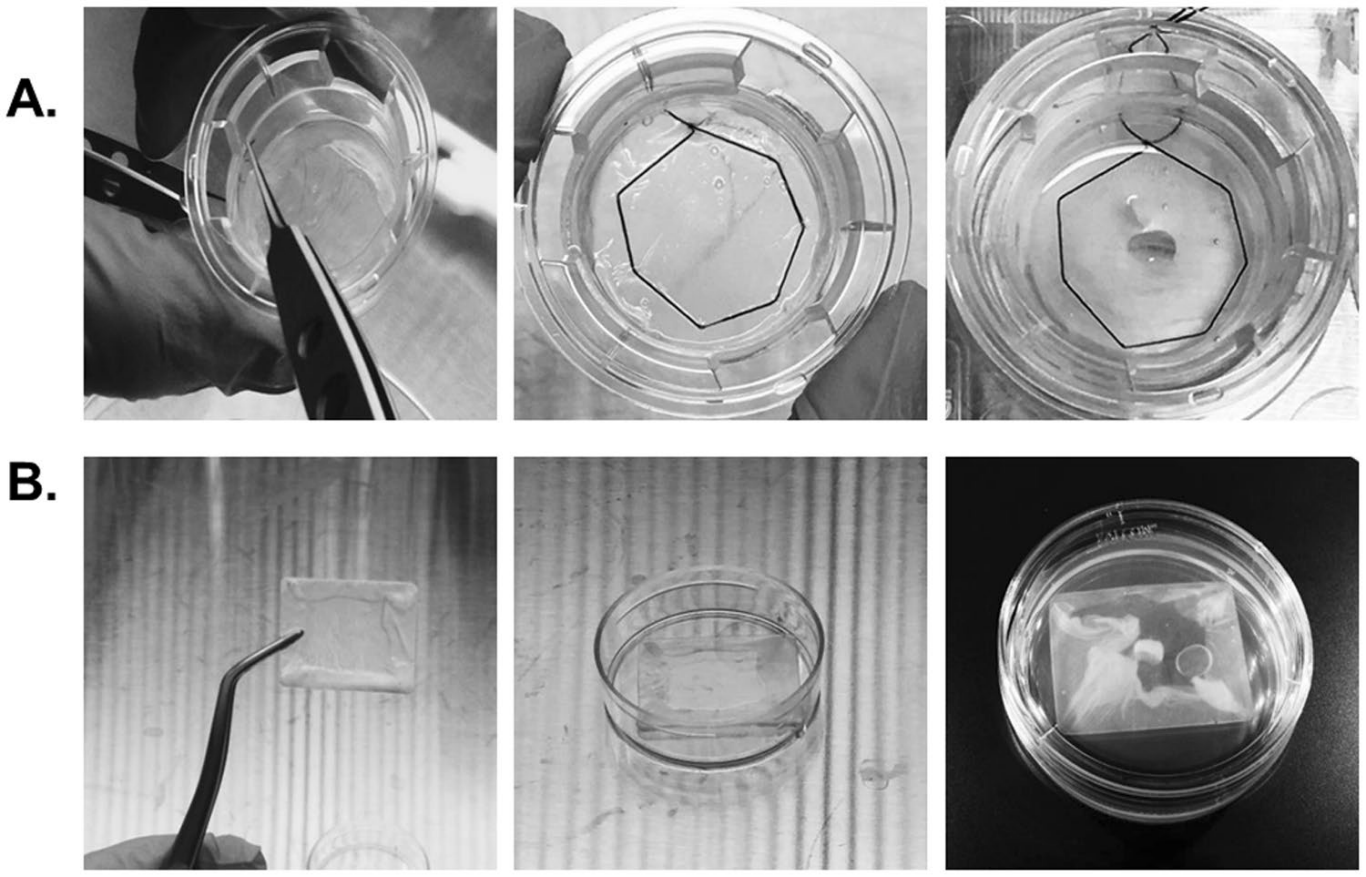
Construction of HAMTW and HAMS. (**A**) HAMTW. HAM was fitted on TW insert and the edge was sutured at 6 points with black silk 6/0 thread in order to form a physical seal and to secure the explant. (**B**) HAMS. Constructed glass slide is enveloped with HAM that is tucked into culture dish.

Culture on Transwell inserts was performed on 25-mm-diameter, 0.4-μm pore polyester membrane inserts (Corning Costar, NY) held in six-well plates. In HAMTW group, de-epithelialized HAM was placed onto Transwell membranes and sutured on six corners as previously published (Fig. 6A)^33,34^. In HAMS group, de-epithelialized HAM was transferred onto the surface of the slide glass (26 × 26 mm) epithelial side facing up so it enveloped the four corners of the slide glass, ensuring there were no wrinkles. The HAMS was tucked into a 35-mm culture dish and pre-equilibrated overnight in culture media (Fig. 6B).

Limbal explants were deposited, epithelial side up, in three conditions: TW, HAMTW, or HAMS and cultured in air–liquid interface conditions. Every 72 h, culture media were refreshed with enough medium to only just cover the explant exposed surface. When outgrowths in control medium reached 70–80% confluence, the limbal epithelial sheets were put in Dispase II (2 mg/ml; Roche, Indianapolis, IN) overnight at 4 °C. CThe outgrown cells were incubated in TrypLE (Gibco) for 10 min to attain fully dissociated cells.

### Clonal proliferation

Cells harvested from limbal epithelial sheets by trypsinization were seeded on collagen type I (PureColl^tm^; Biomatrix, San Diego, CA)–coated 6-well plates at a rate of 100 cells/cm^2^ in CNTP medium (Cell-N-Tec; Bern, Switzerland). CNTP was shown on its preservation of colony-forming efficiency, proliferative capacity, and stem cell-like phenotype of human corneal epithelial cells^38^. Colony formation was monitored daily and analyzed on day 14 after fixation in cold methanol and staining with 0.45% Coomassie blue R 250.

### Flow cytometry

For ABCG2 efflux activity, a property tightly linked to multiple somatic stem cells including the limbal stem cells^39,40^, 2 × 10^4^ cells from limbal explant outgrowth sheets were seeded overnight in SHEM. They were incubated for 45 min with 250 nM JC1 (Axxora, San Diego, CA) and released by a trypsinization for flow cytometry analysis. JC1, a mitochondrial binding dye displaying an accumulation-dependent bathochromic emission shift, is an ABCG2 substratum. In cells displaying high ABCG2, its efflux activity prevents JC1 from reaching its mitochondrial binding sites. Thus, in flow cytometry bivariate 531(green)/585(orange) emission plots, these cells appear as a low-stain cohort (JC1^low^) lying on the left of the cohort of low/nil ABCG2 fully stained cells^27^. Studies were conducted in a FACS Calibur (BD Biosciences, San Diego, CA) instrument.

To stain for p63α (Cell Signaling Technology, Beverly, MA) and CK12 (Santa Cruz Biotechnology, Santa Cruz, CA), cells were (a) enzymatically harvested; (b) fixed with 10% formalin for 10 min; (c) permeabilized with 0.1% Triton X-100 in phosphate-buffered saline (PBS); (d) incubated with 5% BSA for 30 min; (e) incubated with a rabbit polyclonal antibody recognizing p63α or a goat polyclonal antibody recognizing CK12 for 30 min; (f) incubated with Alexa-488–conjugated goat anti-rabbit and rabbit anti-goat IgG (Thermo Fischer, Waltham, MA) for 30 min; and (g) suspended in FACS buffer.

### Western blot

Cells from limbal explant outgrowth sheets were lysed with lysis buffer containing phosphatase inhibitor cocktail 2 (Sigma) and protease inhibitor cocktail (Roche). Proteins of cell lysates were divided in equal amounts by 10% SDS-polyacrylamide gel electrophoresis in reducing conditions and electro-transferred to a PVDF membrane (Millipore, Billerica, MA). The membrane was blocked by 5% skim milk was used, and incubated at 4 °C for 18 h with a mouse monoclonal antibody recognizing ABCG2 (Abcam, Cambridge, MA) or a rabbit polyclonal antibody recognizing p63α. The membranes were washed three times in TBST, and incubated for 1 h at room temperature with the appropriate secondary antibodies conjugated to horseradish peroxidase. After three washes of the membrane, protein bands were distinguished by an enhanced chemiluminescence reagent (ECL; Amersham Biosciences, Piscataway, NJ). All membranes were stripped and reprobed with mouse monoclonal anti-β-actin antibody to provide a normalizing reference. We performed four to six independent experiments and calculated relative levels of expression by image analysis.

### Transmission electron microscopy (TEM)

Limbal epithelial sheets were fixed in 4% paraformaldehdyde and 2.5% glutaraldehyde in 0.1 *M* phosphate buffer for overnight. After a wash in 0.1 M phosphate buffer, the specimens were postfixed with 1% osmium tetroxide in the same buffer for 1 h. The specimens were then dehydrated with a series of graded ethyl alcohols and pure acetone. After the specimens were embedded in Epon 812, polymerization was conducted at 60 °C for 3 days. Ultrathin sections (60~70 nm) were attained by ultramicrotome (Leica Ultracut UCT, Wetzlar, Germany), collected on grids (200 mesh), and examined by TEM (JEM-1010, Jeol, Japan) operating at 60 kV and recorded by CCD camera (SC1000, Gatan, Pleasanton, CA). GMS software (Gatan) was used to measure the length on the electron micrograph.

### Immunofluorescence

When limbal epithelial sheets in control medium reached 70–80% confluence, explants were cut out using a microscalpel, and the outgrown cells on the insert membrane were immune-stained. Cells were fixed with cold methanol, permeabilized with 0.1% Triton X-100, and incubated with 10% goat serum for 1 h to block nonspecific reactions. Then, cells were incubated with the anti- Ki67 (Abcam), p63α and CK15 (Abcam) antibodies, and incubated with Alexa Fluor 488–conjugated anti-rabbit IgG antibody. The stain was captured by confocal microscopy (LSM 510 Meta; Carl Zeiss, Oberkochen, Germany).

### Transplantation of limbal explant outgrowth sheets onto HAMS

Adult New Zealand white rabbits (n = 10) were acquired from Samtako (Osan, Korea). All animals were kept in accordance with the ARVO Statement for the Use of Animals in Ophthalmic and Vision Research. Our study was approved by the Catholic University of Korea Institutional Animal Care and Use Committee.

We used three groups of rabbits for experiments: normal (without injury, n = 2), control (with injury, n = 4), and HAMS transplantation (with HAMS on injury, n = 4). For LSCD injury models, each right eye was subject to application of a paper disk in 0.5 N sodium hydroxide for 30 s. A 360° conjunctival peritomy and surgical limbectomy was conducted. Each limbal explant outgrowth sheet on HAM was lifted from the slide glass and placed on the corneal limbal surfaces with TISSEEL fibrin sealant (Baxter Healthcare, Westlake Village, CA). A therapeutic soft contact lens graft was applied on the graft. Total tarsorrhaphy was performed with 6.0 nylon sutures. For the controls, the same procedures were carried out, except for the transplantation of the limbal explant outgrowth sheet onto HAMS. Postoperatively, the rabbits were treated with topical 0.5% levofloxacin (Samil, Seoul, Korea) twice a day. To inhibit xenogeneic reactions, daily administrations of an intramuscular injection of FK506 (0.2 mg/kg; Astellas, Tokyo, Japan) was done. At 4 weeks after transplantation, corneal fluorescein staining by administration of 0.6 mL of 2.5% fluorescein (Sigma) into the lateral conjunctival sac was performed^27^ and cornea was evaluated after 3 min using portable slit-lamp biomicroscopy under cobalt blue light. Then the rabbits were sacrificed and the corneas were divided into portions for histology and immunohistochemistry.

We deposited 4-μm sections of paraffin-embedded corneas onto silane-coated microscope slides (Matsunami, Osaka, Japan) which were subsequently stained with hematoxylin-eosin or microwaved in Target Retrieval Solution (DAKO, Carpinteria, CA) for 20 mins. The microwaved slides were then washed with phosphate-buffered saline tween (PBST, twice for 10 min) and incubated for 1 h in a 10% BSA blocking buffer. Cells were then incubated with the anti-p63α, CK12, and human nuclei (Abcam) antibodies, and incubated with Alexa Fluor 488 or 546–conjugated anti-rabbit, goat, and mouse IgG antibodies. The stain was captured by confocal microscopy (LSM 510).

### Statistical analysis

Data are expressed as average ± standard deviation. The data were analyzed using a one-way ANOVA where appropriate (SPSS 17.0, Armonk, NY). We regarded p < 0.05 as significant and p < 0.01 highly significant.

## Data Availability

All data from the current study that were generated or analyzed are available upon reasonable request from the corresponding author.
